# Survival trends among non‐small‐cell lung cancer patients over a decade: impact of initial therapy at academic centers

**DOI:** 10.1002/cam4.1749

**Published:** 2018-09-02

**Authors:** Yanyan Lou, Bhagirathbhai Dholaria, Aixa Soyano, David Hodge, Jordan Cochuyt, Rami Manochakian, Stephen J. Ko, Mathew Thomas, Margaret M. Johnson, Neal M. Patel, Robert C. Miller, Alex A. Adjei, Sikander Ailawadhi

**Affiliations:** ^1^ Division of Hematology and Oncology Mayo Clinic Jacksonville Florida; ^2^ Department of Biomedical Statistics and Informatics Mayo Clinic Jacksonville Florida; ^3^ Department of Radiation Oncology Mayo Clinic Jacksonville Florida; ^4^ Department of Cardiovascular Thoracic Surgery Mayo Clinic Jacksonville Florida; ^5^ Division of Allergy and Pulmonary Medicine Mayo Clinic Jacksonville Florida; ^6^ Department of Medical Oncology Mayo Clinic Rochester Minnesota; ^7^ Present address: Department of Blood and Marrow Transplant and Cellular Immunotherapy Moffitt Cancer Center Tampa Florida.

**Keywords:** academic center, community center, National Cancer Database, non‐small‐cell lung cancer, outcome disparities, treatment center type

## Abstract

**Background:**

Treatment of non‐small‐cell lung cancer (NSCLC) has been rapidly advancing over the last decade. Academic centers are considered equipped with better expertise. NSCLC outcome trends in novel therapeutic era and impact of initial treatment at academic centers have not been reported.

**Methods:**

The National Cancer Database (NCDB) was used to identify NSCLC incident cases from 2004 to 2013. Overall survival (OS) was plotted by year of diagnosis and type of initial treatment center, accounting for several factors available in NCDB.

**Results:**

A total of 1 150 722 NSCLC patients were included and separated by initial treatment center type (academic: 31.5%; nonacademic: 68.5%). Median follow‐up and OS for all patients were 11.8 months (range: 0‐133.6 months) and 13.1 months (95% CI: 13.08‐13.17), respectively. Median OS improved significantly for those diagnosed in 2010‐2013 (14.8 months [95% CI: 14.7‐14.9]) as compared to 2004‐2009 (12.4 months [95% CI: 12.3‐12.5]) (*P *<* *0.001). Treatment at academic centers was associated with improved OS (multivariate HR for OS = 0.929 [95% CI: 0.92‐0.94], *P *<* *0.0010). Four‐year OS for academic and nonacademic cohorts was 28.5%% and 22.1%, respectively (*P *<* *0.001), and the difference was more pronounced in stage I to III NSCLC.

**Conclusion:**

In this largest analysis, thus far, NSCLC survival has improved over time, and type of initial treatment center significantly influences survival. Identifying and removing barriers to obtaining initial treatment of NSCLC at academic medical centers could improve OS.

## 1 INTRODUCTION

Lung cancer is the leading cause of cancer‐related mortality in both men and women. It accounts for 13.2% of new cancer cases and 25.9% of all cancer‐related deaths in the United States.^1^ Non‐small‐cell lung cancer (NSCLC) is the most common subtype and accounts for approximately 85% of the lung cancer diagnoses.^2^ Historically, NSCLC is associated with poor survival even when diagnosis is made at early stages due to high risk of micrometastasis despite multimodality treatments. Therapeutic options for NSCLC have increased significantly over the last decade. One of the most important therapeutic advance in lung cancer management had been the identification of specific driver mutations and the development of small molecular tyrosine kinase inhibitors (TKIs).^3^ More recently, checkpoint inhibitors targeting programmed cell death protein 1 (PD‐1) and its ligand (PD‐L1) have been developed, which offer exciting immune‐based therapeutic options. These drugs can achieve durable responses with good tolerability.

Academic centers are at the forefront of these developments with access to clinical trials and advanced diagnostic technologies. Treatment at academic high‐volume centers is associated with improved outcomes of gynecologic cancers, pancreatic cancer, breast cancer, and colon cancer.^4^, ^5^, ^6^ SEER‐Medicare analysis across multiple tumor types has shown 10% reduction in mortality at 1 year for the patients treated at specialty cancer hospitals compared to those at community hospitals.^7^ The National Cancer Database (NCDB) is a prospectively maintained registry covering 70% of newly diagnosed cancer cases including 82% of lung cancer cases with annual follow‐up of at least 90% of the patients.^8^ A previous study by Wang et al analyzing NSCLC outcomes from the NCDB database showed improved outcomes of stage 3 NSCLC treated with concurrent chemoradiation at high‐volume centers.^9^

In this study, we analyzed the survival differences in NSCLC patients who received their initial treatment at academic versus community centers. We hypothesized that initial treatment of patients with NSCLC at academic centers is associated with equal or superior survival compared to those treated at community centers after adjusting for multiple disease‐ and patient‐related factors.

## 2 METHODS

### 2.1 Data source

The NCDB is a hospital‐based national cancer registry created by the American College of Surgeons and American Cancer Society, and it includes an estimated 82% of lung cancers diagnosed nationally. Individual‐level data are entered by professional registrars and are audited.^8^ Participant User File (PUF) for NSCLC was obtained from NCDB for the cases diagnosed from 2004 to 2013.

### 2.2 Study cohorts

The International Classification of Diseases for Oncology, third edition (ICD‐O‐3), codes for histological types of NSCLC were grouped into squamous cell (8052, 8070‐8073, 8076, 8078, 8083, 8084, and 8094), large cell (8012, 8014, 8020, and 8021), adenocarcinoma (8050, 8051, 8140‐8147, 8230, 8250‐8263, 8290, 8310, 8323, 8333, 8470‐8490, and 8550), adenosquamous (8560), sarcomatoid (8022, 8030‐8033, and 8575), and other NSCLCs (8046, 8074, 8075, 8320, 8570, and 8572). Histologies such as carcinoid tumors, other neuroendocrine tumors, and metastatic tumors to the lung were excluded. Cases with occult (N = 2415), stage 0 (N = 2174), and missing treatment center type (N = 8037) were excluded. Final cohort included for analysis was 1 150 722 patients with NSCLC. Academic centers were defined per NCDB treatment center type, code 3: Academic/Research Program (postgraduate medical education in at least four areas and more than 500 newly diagnosed cancer cases per year and includes NCI‐designated comprehensive cancer centers). Facilities which did not meet these criteria were categorized as community centers (code 1: community cancer program; code 2: comprehensive community cancer program; code 4: integrated network cancer program; code 9: other or unknown types of cancer programs). We analyzed the use of adjuvant chemotherapy and radiation in stage I to III NSCLC patients who underwent curative intent primary tumor site surgery. For this analysis, we used site‐specific surgery codes 20‐25 (excision or resection of less than one lobe), 30 (resection of lobe or bilobectomy, but less than the whole lung), 33 (lobectomy with mediastinal lymph node dissection), 45 (lobe or bilobectomy extended, NOS), 46 (lobe or bilobectomy extended with chest wall), 47 (lobe or bilobectomy extended with pericardium), 48 (lobe or bilobectomy extended with diaphragm), 55 (pneumonectomy, NOS), 56 (pneumonectomy with mediastinal lymph node dissection), 65 (extended pneumonectomy), 66 (extended pneumonectomy plus pleura or diaphragm), 70 (extended radical pneumonectomy), 80 (resection of lung, not otherwise specified), and 90 (surgery not otherwise specified). Patients who underwent no primary site surgery were excluded. Patients entered the study on their date of diagnosis and were followed until the most recent date of last contact, death, or the end of the study period, whichever came first. Overall survival (OS) was the primary outcome measure. NCDB captures baseline demographic, clinical data, and information pertaining to the initial management of the respective cancers at the initial treatment center. Data regarding subsequent treatments, site of relapse, time to relapse, or cause of death are not available in NCDB.

### 2.3 Covariates

Mean annual hospital volume of NSCLC cases for each center was calculated. High‐volume facilities were defined as those belonging in the ninetieth percentile of mean annual NSCLC cases rounded to the nearest whole number, with the remainder collective as low volume facilities, as has been used in previous reports.^9^ Demographic factors utilized included age at diagnosis, race (White, Black, Hispanic, Asian/pacific islander, Native American/Alaskan Native), annual median income reported as quartiles for patient's area of residence from 2012 American Community Survey data (<$38 000, $38 000‐$47 999, $48 000‐$62 999, ≥$63 000), age at diagnosis (<60, 60‐69, 70‐79, ≥80), education level reported as percent without high school degree in zip code of patient's primary residence based on American Community Survey data (≥21%, 13%‐20.9%, 7%‐12.9%, <7%), insurance status (no insurance, government insurance, private insurance), geographic region (east coast, central, mountain, pacific), patient location (rural, metropolitan, urban), and travel distance to reporting center (great circle distance, miles). Patient's year of NSCLC diagnosis was used as categorical variable (2004‐2009, 2010‐2013). Clinical factors included Charlson‐Deyo comorbidity score, TNM staging, tumor histology, and type of treatment (radiation, chemotherapy, surgery of primary site, immunotherapy, and palliative care).

### 2.4 Statistical analysis

The distribution of categorical demographic, clinical, and center details was compared between patients treated in academic vs. community centers using Pearson's chi‐square test and Wilcoxon rank sum test, as appropriate. Multivariable logistic regression modeling was used to identify predictors of treatment at academic vs. community centers by calculating odds ratios (OR) for each demographic and clinical variable. Kaplan‐Meier analysis was used to compare median OS between patients treated at academic vs. community centers. Multivariable Cox regression modeling was used as the primary analytic strategy to determine the association between median OS and treatment center type after adjusting for all significant covariates. A two‐sided *P‐*value of less than 0.05 was used to determine statistical significance. Confidence interval (CI) limit was set at 95%.

## 3 RESULTS

A total of 115 0722 NSCLC patients were included in the analysis with median follow‐up of 11.8 months (range 0‐133.6 months). A total of 362 247 (31.5%) NSCLC patients were treated at academic centers and 788 475 (68.5%) at community centers with median follow‐up of 14.1 months (range 0‐133.6 months) and 10.8 months (range 0‐131.7 months), respectively. Table 1 demonstrates baseline patient demographic and clinical parameters by treatment center type. On logistic regression model, patients treated at academic centers were significantly more likely to be younger, non‐White, carrying private insurance, and living in metropolitan area with higher annual income and education. They also travelled farther to their treatment center. Median travel distance between patient's primary residence and treatment center was greater for patients treated at academic centers (11.2 vs 8.1 miles, *P *<* *0.0001). These patients scored low on Charlson‐Deyo comorbidity score and had lower TNM stage. All of these variables were significantly different with *P *<* *0.0001. Academic centers were more likely to be high‐volume facilities (OR = 4.64, 95% CI: 4.58‐4.69, *P *<* *0.0001, Table S1).

**Table 1:** Baseline demographics and clinical parameters of patients with non‐small‐cell lung cancer by type of treatment facility

	Academic center (N = 362 247)	Community center (N = 788 475)	*P*‐value
Median age at diagnosis	68.0 (40‐90)	70.0 (40‐90)	<0.0001
Race			
White	289 097 (80.8%)	696 610 (88.9%)	<0.0001
Black	55 564 (15.5%)	69 055 (8.8%)	
Asian/Pacific Islander	10 227 (2.9%)	13 313 (1.7%)	
Hispanic	2153 (0.6%)	2457 (0.3%)	
Other	543 (0.2%)	2147 (0.3%)	
Missing	4663	4893	
Median annual income quartiles			
<$38 000	77 385 (21.8%)	155 164 (20.2%)	<0.0001
$38 000‐$47 999	78 906 (22.2%)	209 503 (27.2%)	
$48 000‐$62 999	87 802 (24.7%)	211 346 (27.4%)	
$63 000+	110 998 (31.3%)	194 024 (25.2%)	
Missing	7156	18 438	
Percent without high school degree			
≥21%	70 258 (19.8%)	138 128 (17.9%)	<0.0001
13%‐20.9%	95 988 (27.0%)	223 792 (29.0%)	
7%‐12.9%	109 041 (30.7%)	259 726 (33.7%)	
<7%	79 966 (22.5%)	148 814 (19.3%)	
Missing	6994	18 015	
Insurance status			
No insurance	13 054 (3.7%)	11 758 (1.5%)	<0.0001
Government insurance	228 900 (65.6%)	546 382 (71.4%)	
Private insurance	106 864 (30.6%)	206 993 (27.1%)	
Missing	13 429	23 342	
Type of area			
Metropolitan	304 248 (86.8%)	599 865 (79.3%)	<0.0001
Urban	41 431 (11.8%)	136 502 (18.0%)	
Rural	4737 (1.4%)	20 119 (2.7%)	
Missing	11 831	31 989	
Geographic region			
East Coast	180 148 (49.7%)	312 061 (39.6%)	<0.0001
Central	149 153 (41.2%)	352 437 (44.7%)	
Mountain	9658 (2.7%)	33 572 (4.3%)	
Pacific	23 288 (6.4%)	90 405 (11.5%)	
Charlson‐Deyo comorbidity score			
0	228 517 (63.1%)	453 021 (57.5%)	<0.0001
1	93 742 (25.9%)	232 322 (29.5%)	
≥2	39 988 (11.0%)	103 132 (13.1%)	
Year of diagnosis			
2004‐2009	204 897 (56.6%)	465 401 (59.0%)	<0.0001
2010‐2013	157 350 (43.4%)	323 074 (41.0%)	
TNM stage			
Stage 1	83 398 (27.8%)	157 977 (24.1%)	<0.0001
Stage 2	19 881 (6.6%)	45 890 (7.0%)	
Stage 3	68 832 (23.0%)	160 407 (24.5%)	
Stage 4	127 558 (42.6%)	290 783 (44.4%)	
Missing	62 578	133 418	
Radiation therapy			
No	216 768 (60.4%)	463 613 (59.5%)	<0.0001
Yes	141 842 (39.6%)	316 214 (40.5%)	
Missing	3637	8648	
Surgery of primary site			
No	235 121 (65.1%)	572 067 (72.8%)	<0.0001
Yes	126 128 (34.9%)	213 605 (27.2%)	
Missing	998	2803	
Chemotherapy			
No	197 689 (56.6%)	423 392 (55.3%)	<0.0001
Yes	151 604 (43.4%)	341 973 (44.7%)	
Missing	12 954	23 110	
Immunotherapy			
No	357 575 (99.5%)	779 369 (99.6%)	<0.0001
Yes	1747 (0.5%)	3158 (0.4%)	
Missing	2925	5948	
Palliative care			
No	316 203 (88.5%)	696 762 (88.4%)	0.6659
Yes	41 233 (11.5%)	91 106 (11.6%)	
Missing	4811	607	
High‐volume facility			
No	169 003 (46.7%)	633 425 (80.3%)	<0.0001
Yes	193 244 (53.3%)	155 050 (19.7%)	
Median distance from treatment facility (miles)	11.2	8.1	<0.0001
Median overall survival (Months)	16.4	11.9	<0.0001

**Figure 1:**
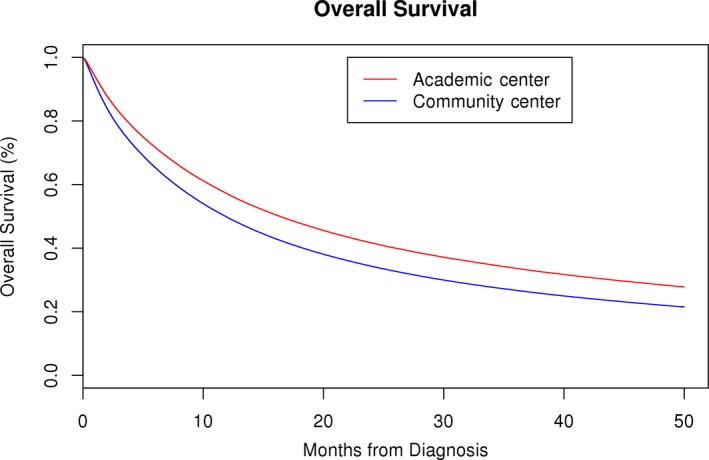
Kaplan‐Meier survival curve of non‐small‐cell lung cancer patients who received initial treatment at academic and community centers (log‐rank hazard ratio for overall survival—0.819, *P *<* *0.001)

Median OS for the entire cohort was 13.1(95% CI: 13.08‐13.17) months. Median OS of patients treated at academic centers and community centers was 16.4 months (95% CI: 16.2‐16.5 months) and 11.9 months (95% CI: 11.8‐11.9 months), respectively. Four‐year OS of patients treated at academic centers and community centers was 28.5% (95% CI: 28.3‐28.7%) and 22.1% (95% CI: 22.0‐22.2%), respectively (Figure 1). Log‐rank hazard ratio (HR) for OS of the patients treated at academic centers was 0.82 (95% CI: 0.82‐0.82) (*P *<* *0.001). After controlling for demographic and clinical factors in Cox proportional hazards modeling, treatment at academic center was independently associated with a decreased hazard of death (HR = 0.93; 95% CI: 0.92‐0.93; *P *<* *0.0001). Other factors associated with improved OS were younger age, Asian and Hispanic race/ethnicity, private insurance, higher education, higher median annual household income, low TNM stage, and low Charlson‐Deyo comorbidity score (Table 2). Patients diagnosed between the years 2010 and 2013 had better survival compared to the patients who were diagnosed between the years 2004 and 2009 (HR = 0.92 95% CI: 0.91‐0.92, *P *<* *0.0001, Figure S1). There was an improvement in 4‐year OS over time (2004‐2009 = 23.4% [95% CI: 23.3%‐23.5%] vs 2010‐2013 = 25.2% [95% CI: 25%‐25.4%] *P *<* *0.001). OS analysis by major histological subtypes of NSCLC was performed between academic and community centers. HRs of OS for adenocarcinoma, squamous cell carcinoma, and other histologies (large cell, sarcomatoid, and other NSCLC) were 0.79 (95% CI: 0.79‐0.80), 0.86 (95% CI: 0.85‐0.87), and 0.90(95% CI: 0.89‐0.91), respectively (Figure S2).

**Table 2:** Cox proportional hazards multivariable model for predictors of overall survival of patients with non‐small‐cell lung cancer

Parameter	Hazard Ratio	95% Hazard ratio confidence limits	*P*‐value
Facility type (Community center)			
Academic center	0.929	0.922‐0.936	<0.0001
Age at diagnosis (<60)			
60‐69	1.06	1.05‐1.07	<0.0001
70‐79	1.171	1.159‐1.182	<0.0001
≥80	1.304	1.289‐1.319	<0.0001
Race (white)			
Black	0.946	0.936‐0.956	<0.0001
Asian/Pacific Islander	0.784	0.765‐0.802	<0.0001
Hispanic	0.811	0.767‐0.858	<0.0001
Native American/Alaska Native	0.953	0.895‐1.015	0.1329
Charlson‐Deyo comorbidity score(0)			
1	1.182	1.174‐1.19	<0.0001
≥2	1.379	1.366‐1.392	<0.0001
Median local annual income(˂$38 000)			
$38 000‐$47 999	0.975	0.966‐0.984	<0.0001
$48 000‐$62 999	0.965	0.955‐0.975	<0.0001
$63 000+	0.929	0.918‐0.941	<0.0001
Percent without high school diploma (≥21%)			
13‐20.9%	1.013	1.003‐1.023	0.0084
7‐12.9%	1.005	0.995‐1.016	0.3186
<7%	0.975	0.962‐0.987	<0.0001
Insurance status (Uninsured)			
Government insurance	0.962	0.938‐0.987	0.0027
Private insurance	0.868	0.846‐0.891	<0.0001
Type of residential area (Metropolitan)			
Urban	1.015	1.006‐1.024	0.0008
Rural	1.028	1.007‐1.049	0.0094
Geographic region (East coast)			
Central	1.059	1.052‐1.066	<.0001
Mountain	0.969	0.953‐0.986	0.0003
Pacific	0.981	0.971‐0.992	0.0008
Year of diagnosis (2004‐2009)			
2010‐2013	0.876	0.871‐0.882	<0.0001
TNM stage (stage 1)			
Stage 2	1.86	1.835‐1.886	<0.0001
Stage 3	2.462	2.437‐2.489	<0.0001
Stage 4	4.183	4.14‐4.228	<0.0001
Distance from treatment facility(per 500 mile change)	0.924	0.906‐0.942	<0.0001
Facility volume (Low volume)			
High‐volume center	0.951	0.944‐0.958	<0.0001
Chemotherapy (No)			
Yes	0.550	0.547‐0.554	<0.0001
Immunotherapy (No)			
Yes	0.774	0.728‐0.822	<0.0001
Palliative care (No)			
Yes	1.370	1.358‐1.383	<0.0001
Radiation (No)			
Yes	0.903	0.897‐0.909	<0.0001
Surgery (No)			
Yes	0.404	0.399‐0.409	<0.0001

**Figure 2:**
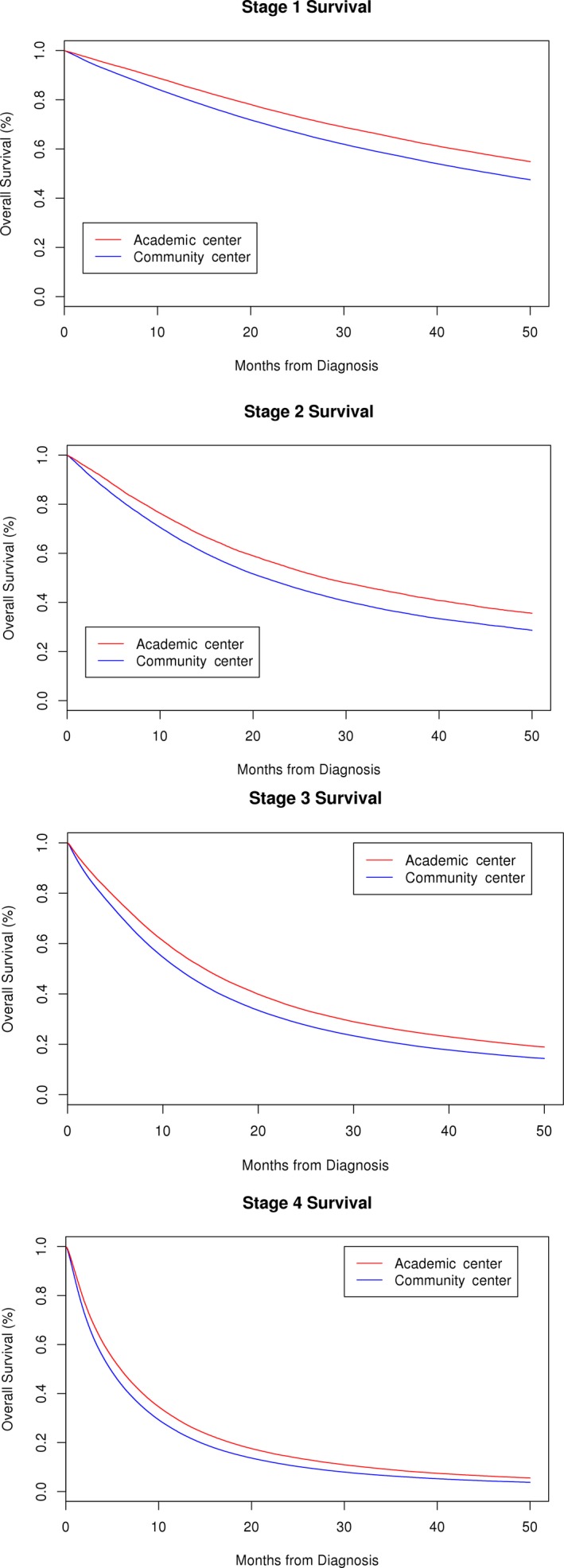
Kaplan‐Meier survival curves of stage 1‐4 non‐small‐cell lung cancer patients who received initial treatment at academic and community center (*P *<* *0.001)

We subsequently calculated Log‐rank HR for OS by each NSCLC stage between academic vs. community centers (Table 3, Figure 2). OS was better in academic centers across all TNM stages (*P *<* *0.001). The improvement in survival was more pronounced in early stages of NSCLC as evident by HR for OS for stage 1 being 0.80 (95% CI: 0.79‐0.81, *P *<* *0.0001) and for stage 4 being 0.86 (95% CI: 0.85‐0.87, *P *<* *0.0001). Difference in 4‐year OS between academic and community centers for stage 1 was 56.0% (95% CI: 55.6‐56.5%, *P *<* *0.0001) vs. 48.7% (95% CI: 48.4‐49.0%, *P *<* *0.0001) and for stage 4 was 5.9% (95% CI: 5.7‐6.0%, *P *<* *0.0001) vs. 4.0% (95% CI: 3.9‐4.1%, *P *<* *0.0001).

**Table 3:** Overall survival of non‐small‐cell lung cancer patients by disease stage between academic vs community center

	Academic Center				Community Center					*P*‐value
TNM stage	Number of patients	Median OS (months)	4‐year OS (%)	95% CI for OS	Number of patients	Median OS (months)	4‐year OS	95% CI for OS	Log‐rank hazard ratio	
1	70 990	58.7	56.0	55.6‐56.5	137 026	46.0	48.7	48.4‐49.0	0.796	<0.00001
2	16 784	27.7	36.4	35.6‐37.3	39 320	21.3	29.6	29.0‐30.1	0.809	<0.00001
3	61 678	14.4	19.6	19.2‐19.9	144 763	11.6	14.9	14.7‐15.1	0.841	<0.00001
4	111 697	5.9	5.9	5.7‐6.0	256 797	4.8	4.0	3.9‐4.1	0.861	<0.00001
Stage missing	59 362	35.9	44.2	43.7‐44.6	128 421	21.9	35.0	34.8‐35.3	0.777	<0.00001
Total^a^	320 511	16.4	28.5	28.3‐28.7	706 327	11.9	22.1	22.0‐22.2	0.819	<0.00001

Total number of patients is less than original cohort due to missing data on OS for 123 884 patients.

We also looked at the difference in treatment patterns between patients treated at academic and community centers. Rate of surgery in overall studied patients was significantly higher at academic centers compared to community centers as shown in Table 1 (34.9% vs 27.2%, *P *<* *0.0001). To understand the role of curatively intended primary surgery, we selected stages I, II and III NSCLC who underwent primary site surgery and received adjuvant chemotherapy and/or radiation (Figure S3). Table 4 shows types and rate of surgery and rate of adjuvant chemotherapy and radiation therapy use in nonmetastatic NSCLC patients by treatment center. More lobectomy, less wedge resection and segmental resection were performed at community center than academic center. Overall use of adjuvant therapy was quite similar between academic and community centers (30.1% vs 30.8%, *P = *0.0003), with slightly more patients treated at community centers receiving adjuvant radiation therapy compared to academic centers (12.5% vs 13.7%, *P *<* *0.0001). Rate of adjuvant chemotherapy was similar between the groups (28.1% vs 28.6%, *P *=* *0.014). Next, we focused on nonsurgically managed patients who received radiation therapy to primary tumor site. This analysis was limited to the stage I‐III patients who did not received surgery of primary tumor site. Significantly more stage I NSCLC patients from academic center received radiation therapy compared to community center (71.2% vs 61.5% *P *<* *0.001, Table S2). There was no difference in the use of primary tumor site radiation for stage II (*P *=* *0.87) and III (*P = *0.07) NSCLC patients between the two center types (Table S2). There was a statistically significant difference in the rates of chemotherapy, immunotherapy, radiation therapy, and palliative care between academic and community centers; however, the difference was quite small and unlikely to be clinically meaningful (Table 1).

**Table 4:** Type of primary tumor site surgery and adjuvant therapies in stage I, II and III non‐small‐cell lung cancer patients treated at academic and community centers

Primary surgery and adjuvant therapy	Academic center (N = 79 510)	Community center (N = 136 643)	Total (N = 216 153)	*P*‐value
Excision or Resection of less than one Lobe	18 109 (22.8%)	28 413 (20.8%)	46 522 (21.5%)	<0.0001
Lobectomy or bilobectomy	55 741 (70.1%)	100 041 (73.2%)	155 782 (72.1%)	<0.0001
Pneumonectomy	3892 (4.9%)	7133 (5.2%)	11 025 (5.1%)	0.0009
Surgery not otherwise specified	1768 (2.2%)	1056 (0.8%)	2824 (1.3%)	<0.001
No adjuvant therapy	55 148 (69.9%)	93 745 (69.2%)	148 893 (69.5%)	0.0003
Adjuvant chemotherapy	21 429 (28.1%)	37 751(28.6%)	59 180 (28.4%)	0.0142
Adjuvant radiation therapy	9773 (12.5%)	18 496 (13.7%)	28 269 (13.3%)	<0.0001
Missing chemotherapy information	3150	4488	7638	
Missing radiation information	1016	2012	3028	
Missing adjuvant therapy information	663	1149	1812	

Stage distribution: stage 1 = 155 256 (71.8%); stage 2 = 31 201 (14.4%); stage 3 = 29 696 (13.7%).

## 4 DISCUSSION

Therapeutic advances in cancer management have undoubtedly led to overall improvement in outcomes and patient survival. Nevertheless, disparities exist in outcomes of patients based on several variables, some of which are modifiable. In this largest analysis of NSCLC patients thus far, treatment at academic centers was associated with improved OS compared to community centers. This survival difference was greater in nonmetastatic NSCLC. This analysis also shows improvement in OS over time with the patients diagnosed between the years 2010 and 2013 had better OS compared to those who were diagnosed between the years 2004 and 2009 (median OS: 12.4 vs 14.6 months *P *<* *0.0001). This trend shows the real‐world impact of recent developments in NSCLC management, likely related to improvement in early detection, advanced surgical and radiation techniques, development of targeted drugs, and routine incorporation of adjuvant chemotherapy in surgically resected nonmetastatic NSCLC.

These results are consistent with previous smaller reports of NSCLC outcomes and impact of treatment center type. Meguide et al analyzed surgical outcomes utilizing the Nationwide Inpatient Sample (NIS) dataset and showed that in‐hospital mortality after lung cancer resection was less at teaching hospitals and higher‐volume centers (adjusted OR: 0.83, 95% CI: 0.73‐0.93, *P *=* *0.002).^10^ In a separate study based on Florida Cancer Data System, patients undergoing curative intent lung cancer resection at teaching centers and high‐volume centers had improved 30‐day (2.7% vs 1.6%, *P *<* *0.001), 90‐day (7.5% vs 4.0%, *P *<* *0.001), and 5‐year (63.5% vs 59.3%, *P *=* *0.002) mortality.^11^ Samson et al showed lower 30‐day mortality of stage 3A NSCLC patients treated at academic centers based on NCDB dataset 1998‐2010.^12^ Quality of surgery is also influenced by hospital case volume. Analysis of lung cancer patients undergoing lobectomy or pneumonectomy from the national Medicare database (1998‐1999) showed operative mortality was lowest for the patients operated by noncardiac thoracic surgeon with high case volume.^13^ Radiation therapy (RT) is associated with improved local control and OS compared to no‐active treatment for stage I NSCLC patients.^14^ In our analysis, nonsurgically managed stage I patients were more likely to receive primary tumor site radiation therapy at academic centers compared to community centers.

Management of NSCLC is increasingly becoming complex with routine use of endobronchial ultrasound (EBUS) for mediastinal staging, mutation testing for targeted therapies, as well as adjuvant chemotherapy and radiation in some cases. National Comprehensive Cancer Network (NCCN) recommends multidisciplinary evaluation of suspected stage 1‐3 lung cancer for optimal diagnostic evaluation and management.^15^ A review of stage 3A, 3B, or 4 NSCLC patients from SEER‐Medicare database and American Medical Association Masterfile database showed that patients who were referred to medical oncologists, radiation oncologists, and surgeons had higher likelihood of treatment adherence to NCCN guidelines.^16^ Adjuvant chemotherapy is associated with improved survival of surgically resected high‐risk nonmetastatic NSCLC.^17^, ^18^ The benefit of postoperative radiotherapy (PORT) remains uncertain. PORT was associated with worse outcomes in patients with early‐stage NSCLC in a meta‐analysis, but several studies have also indicated patients with pN2 disease or incomplete resection may benefit from PORT.^19^, ^20^, ^21^ A SEER analysis revealed wide variation in the use of adjuvant chemotherapy and radiation in eligible patients, which remained significant even after adjusting for patient‐ and area‐level covariates.^22^ In our analysis, difference in use of adjuvant therapy was nominal between academic and community centers for stage I‐III patients with slightly increased use of adjuvant radiation therapy at community centers.

Academic centers are often associated with research facilities and have broader access to clinical trials for their patients. A single institute study of 78 resected NSCLC patients enrolled in prospective, randomized investigational trials from Fred Hutchinson Cancer Center demonstrated better survival compared to population‐based control group of patients not included in such trials.^23^ A similar study in small cell lung cancer showed improved survival of protocol patients compared to nonprotocol patients, which remained significant even after adjusting for patient factors.^24^ However, this “trial‐effect” was not detected in another study by Abu‐Hejleh et al These investigators studied 815 stage 3B or 4 NSCLC patients from the Cancer Care Outcomes Research and Surveillance Consortium (CanCORS) and showed that patients treated within clinical trial setting conveyed better perception of quality of care but did not demonstrate a survival benefit (*P *=* *0.21).^25^ As NCDB does not provide information about participation in clinical trials, we are unable to determine its impact on survival. However, we hypothesize that greater alignment with protocol‐based treatment at academic centers may be casually linked to improved OS for early TNM stage patients in our study.

There was a difference in several demographic and socioeconomic factors between the patients receiving initial therapy for NSCLC at academic vs. community centers. Patients treated at academic centers were more likely to be Black (African American), Asian, or Hispanic. Asians with NSCLC have improved OS due to difference in disease biology such as increased prevalence of epidermal growth factor receptor (EGFR) mutation.^26^, ^27^ A California Cancer Registry study showed improved NSCLC‐specific mortality among foreign‐born Hispanics compared to non‐Hispanic Whites and Hispanics born in United States.^28^ In our study, Asian race and Hispanic race were associated with improved OS in multivariate model with the HR for OS being 0.78 and 0.81, respectively (*P *<* *0.001). Besides race, academic centers also served more privately insured, educated, higher income patients residing in metropolitan areas. Patients with more resources tend to travel farther to seek better care at larger referral centers, which are frequently academic and are more likely to be in the urban metropolitan areas.

To the best of our knowledge, this is the largest analysis to date comparing survival of NSCLC across all stages by treatment center type. We looked at range of socioeconomic factors, insurance status, and treatment modalities and adjusted in multivariate fashion to compare OS difference between academic and community centers. The patients were diagnosed and received initial treatment at the same facility. In our opinion, this strategy minimized the referral bias for academic centers. Additionally, these data revealed an improvement in OS of NSCLC over time (2004‐2009 vs 2010‐2013) in a population‐based database.

This study is limited by being a retrospective review of prospectively collected data and the absence of certain data points in the NCDB database. Patient's performance status is an important factor affecting survival and selection of therapy that is not included in the NCDB dataset. To account for the absence of information on performance status, we used the Charlson‐Deyo comorbidity score, which is available in the dataset, as a surrogate characteristic to stratify patients.

Another limitation of our study is the potential for selection bias as only patients treated at Commission on Cancer (CoC) designated programs are included in NCDB database. A comparison of CoC‐approved and non‐CoC‐approved hospitals based on American Hospital Association Annual Survey Database (2006) showed that CoC‐approved programs were more likely to be larger facilities with more cancer‐related services and located in urban locations.^29^ Annual household income and education were estimated based on patient's primary residential area, as captured in the NCDB database, and not individual patient‐reported information. NCDB only reports on the initial treatment for a given patient and, subsequent therapy information is not captured. Information about disease relapse and treatment toxicities and details about systemic chemotherapeutic agents are not reported. A significant number of patients have missing data, which could be used in the Cox proportional regression analysis. However, because we have such a large proportion of patients with completed data, confidence intervals of the reported HRs in the multivariate regression models are quite narrow, and thus, it is doubtful that availability of missing data would change HR. However, detailed single patient analysis of multi‐institutional studies across the disease spectrum is needed to verify our findings. Such detailed data may also be available from claims‐based large datasets, for example SEER‐Medicare, OPTUM, Truven data. Meanwhile, cancer center registry‐based studies like this one can help define disparities in NSCLC outcomes between different treatment facilities and highlight relevant patient‐ and disease‐related factors.

## 5 CONCLUSIONS

This large analysis shows that treatment of patients with NSCLC at academic centers is associated with improved OS compared with those treated at community centers. The difference was more pronounced among nonmetastatic NSCLC, compared to metastatic NSCLC patients. These findings strongly support referring NSCLC patients to academic facilities with multidisciplinary expertise. Changes in policy and healthcare infrastructure are mandatory in order to facilitate patient's access to academic cancer centers. Community cancer facilities remain a very important resource for cancer care in this country. These faculties must be provided with resources to seamlessly partner with academic medical centers, improve quality of care, and enhance access to clinical trials. Greater collaboration between academic and community centers is critical to improve access to specialty care most especially for the socioeconomically disadvantaged patients and those in rural areas.

## CONFLICT OF INTEREST

None of the authors have any conflict of interest.
