# Supplementary figures and images for: Survival trends among non‐small‐cell lung cancer patients over a decade: impact of initial therapy at academic centers
Source: Cancer Med. 2018 Sep 2;7(10):4932–42. doi: 10.1002/cam4.1749 (PMC6198232; doi:10.1002/cam4.1749)

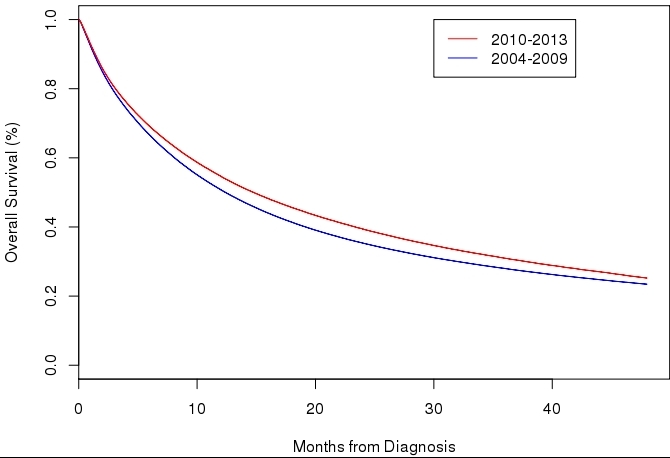

Supplement: Supplementary file 1 [file CAM4-7-4932-s001.tif]

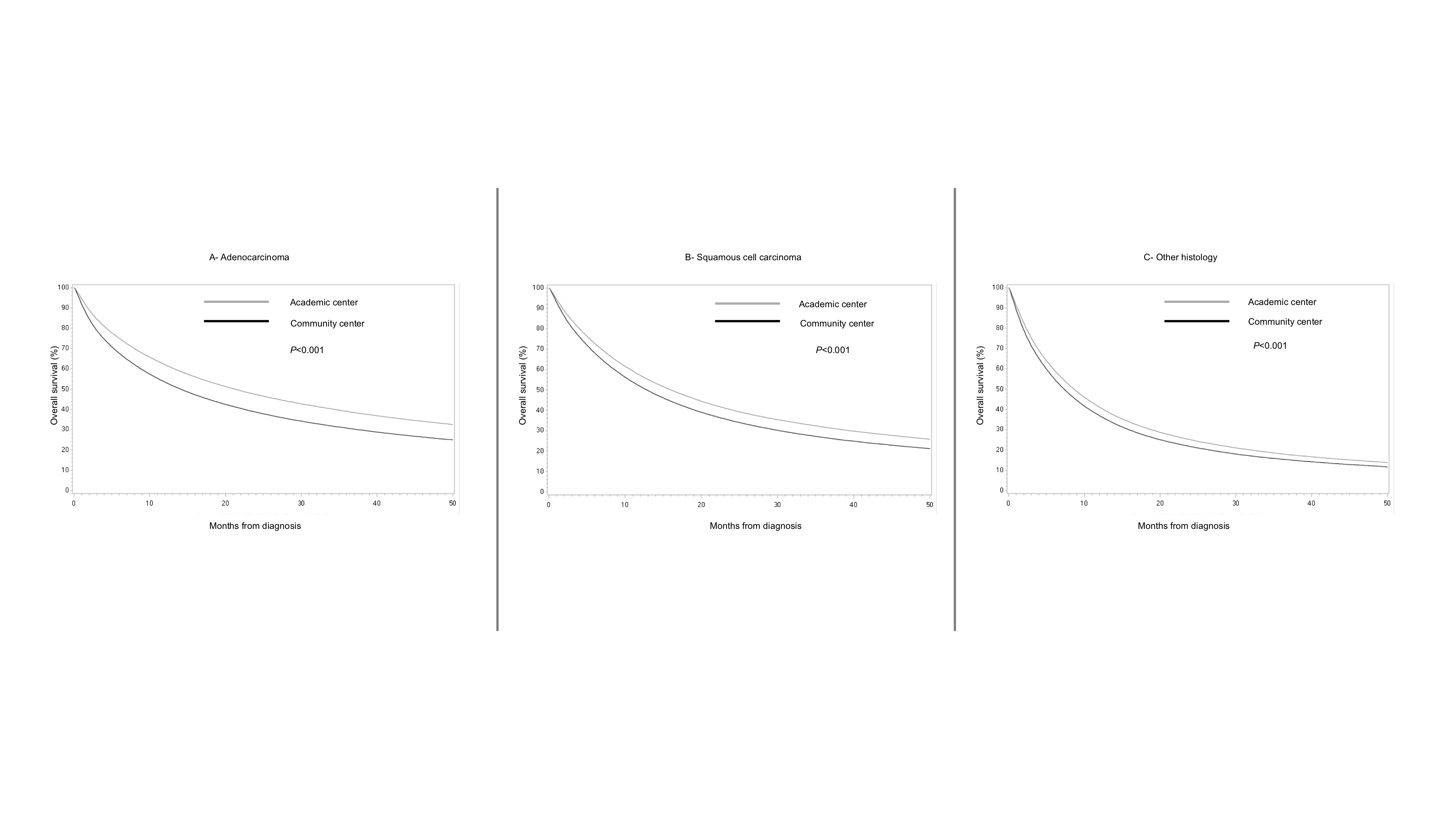

Supplement: Supplementary file 2 [file CAM4-7-4932-s002.tiff]

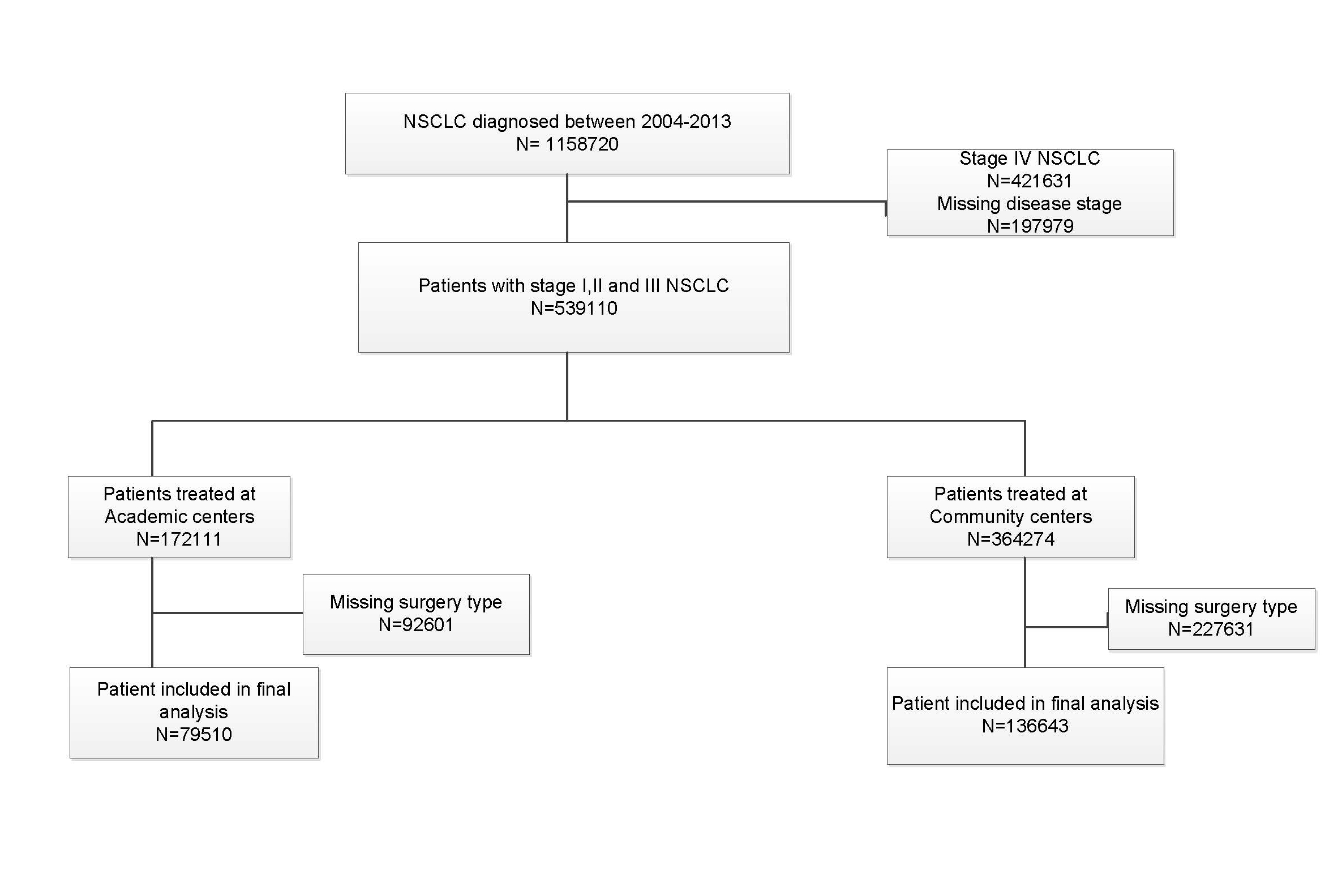

Supplement: Supplementary file 3 [file CAM4-7-4932-s003.jpg]
