# Supplementary material for: Survival trends among non‐small‐cell lung cancer patients over a decade: impact of initial therapy at academic centers
Source: Cancer Med. 2018 Sep 2;7(10):4932–42. doi: 10.1002/cam4.1749 (PMC6198232; doi:10.1002/cam4.1749)
Supplement: Supplementary file 4 [file CAM4-7-4932-s004.docx]

Supplementary table S1: Logistic Regression Model Estimating Factors Associated with Treatment at Academic Center

| **Characteristic (reference)** |  | **Odd Ratio** | **95% Confidence Interval** | | ***p*-value** |
| --- | --- | --- | --- | --- | --- |
| Facility volume (low-volume) | High-volume | 4.637 | 4.583 | 4.692 | <.0001 |
| TNM stage (stage 1) | Stage 2 | 0.915 | 0.893 | 0.936 | <.0001 |
|  | Stage 3 | 1.009 | 0.990 | 1.029 | 0.3301 |
|  | Stage 4 | 1.041 | 1.022 | 1.061 | <.0001 |
| Age at diagnosis in years (18-59) | 60-69 | 0.870 | 0.855 | 0.884 | <.0001 |
|  | 70-79 | 0.758 | 0.744 | 0.771 | <.0001 |
|  | >=80 | 0.697 | 0.682 | 0.713 | <.0001 |
| Race (White) | Hispanic (all races) | 1.753 | 1.603 | 1.918 | <.0001 |
|  | Asian/Pacific Islander | 2.181 | 2.097 | 2.268 | <.0001 |
|  | Black | 1.884 | 1.849 | 1.919 | <.0001 |
|  | Native American/Alaska Native | 0.901 | 0.789 | 1.029 | 0.1243 |
| Median local annual income (<$38,000) | $38,000-$47,999 | 0.903 | 0.887 | 0.921 | <.0001 |
|  | $48,000-$62,999 | 0.981 | 0.961 | 1.001 | 0.0628 |
|  | $63,000+ | 1.351 | 1.319 | 1.383 | <.0001 |
| Percent without high school diploma (≥21%) | 13-20.9% | 0.857 | 0.842 | 0.873 | <.0001 |
|  | 7-12.9% | 0.816 | 0.800 | 0.833 | <.0001 |
|  | <7% | 0.872 | 0.851 | 0.894 | <.0001 |
| Insurance statue (uninsured) | Government Insurance | 0.620 | 0.593 | 0.649 | <.0001 |
|  | Private insurance | 0.635 | 0.606 | 0.664 | <.0001 |
| Type of residential area (Metro) | Rural | 0.480 | 0.459 | 0.503 | <.0001 |
|  | Urban | 0.636 | 0.625 | 0.648 | <.0001 |
| Distance from treatment facility(per 500 mile change) | | 3.009 | 2.883 | 3.142 | <.0001 |
| Geographic region (central) | East Coast | 1.245 | 1.230 | 1.260 | <.0001 |
|  | Mountain | 0.703 | 0.679 | 0.729 | <.0001 |
|  | Pacific | 0.676 | 0.661 | 0.692 | <.0001 |
| Year of diagnosis (2004-209) | 2010-2013 | 1.080 | 1.068 | 1.093 | <.0001 |
| Charlson-Deyo comorbidity score(0) | 1 | 0.794 | 0.783 | 0.804 | <.0001 |
|  | ≥2 | 0.805 | 0.791 | 0.820 | <.0001 |
| Chemotherapy use (No) | Yes | 0.899 | 0.887 | 0.911 | <.0001 |
| Immunotherapy use (No) | Yes | 1.130 | 1.041 | 1.227 | 0.0037 |
| Palliative therapy use (No) | Yes | 0.988 | 0.969 | 1.007 | 0.2281 |
| Radiation therapy use (No) | Yes | 1.087 | 1.072 | 1.101 | <.0001 |
| Surgery at primary tumor site (No) | Yes | 1.384 | 1.360 | 1.408 | <.0001 |
