# Supplementary material for: Survival trends among non‐small‐cell lung cancer patients over a decade: impact of initial therapy at academic centers
Source: Cancer Med. 2018 Sep 2;7(10):4932–42. doi: 10.1002/cam4.1749 (PMC6198232; doi:10.1002/cam4.1749)
Supplement: Supplementary file 5 [file CAM4-7-4932-s005.rtf]

Supplementary Table S2: Use of Primary Tumor-site Radiation in non-metastatic Non-Small Cell Lung Cancer Patients Who Did Not Underwent Primary Tumor Site Surgery.


Use of radiation therapy as primary local treatment	Missing facility type	Academic center	Community center	P-value	
Stage 1		24672	55936	<0.0001	
Yes	19	17559 (71.2%)	34374 (61.5%)		
No	60	7111 (28.8%)	21542 (38.5%)		
Missing information on radiation therapy	0	2	20		
					
Stage 2					
Yes	28	5029 (62.5%)	15214 (62.6%)	0.8679	
No	35	3021 (37.5%)	9099 (37.4%)		
Missing information on radiation therapy	0	3	5		
					
Stage 3				0.0741	
Yes	508	32134 (61.2%)	82122 (61.6%)		
No	321	20410 (38.8%)	51184 (38.4%)		
Missing information on radiation therapy	0	10	35		
